# Smart Toothbrushes and AI-Assisted Oral Hygiene Monitoring for Plaque Control, Gingival Health, and Periodontal Care: A Narrative Review

**DOI:** 10.3390/dj14100672

**Published:** 2026-10-14

**Authors:** Ghadah K. Bahamdan

**Affiliations:** Department of Oral Diagnosis, Oral Biology and Periodontology, College of Dentistry, King Khalid University, Abha 61421, Saudi Arabia; ghbahamdan@kku.edu.sa

**Keywords:** smart toothbrush, artificial intelligence, oral hygiene, plaque control, gingivitis, periodontitis, telemonitoring

## Abstract

Effective toothbrushing is central to plaque control and periodontal health, yet brushing technique and adherence are often inadequate. Smart toothbrushes and digitally assisted oral hygiene systems record brushing behaviour and return feedback to patients and, in some models, to clinicians. This narrative review, prepared with reference to the SANRA checklist, drew on PubMed/MEDLINE, Scopus, Web of Science, the Cochrane Library, and Google Scholar using a targeted, non-systematic search for publications from January 2012 to February 2026. Eighteen primary studies are summarised in detail. Sensor-based and app-connected systems improved plaque scores, gingival bleeding, brushing behaviour, and, in some studies, adherence, mostly over follow-up periods of 2 weeks to 6 months. Two randomised trials in patients with periodontitis reported additional short-term improvements in periodontal parameters when AI-assisted monitoring was added to periodontal care. However, many commercial devices use fixed thresholds rather than adaptive machine learning, and most trials cannot separate the effects of powered brushing, feedback, app support, and AI. Current evidence suggests that smart toothbrushes and digitally assisted oral hygiene systems may improve plaque control, brushing behaviour, adherence, and short-term gingival outcomes. The incremental benefit specifically attributable to AI-based functions and their impact on long-term periodontal outcomes remain to be established.

## 1. Introduction

**Figure 1:**
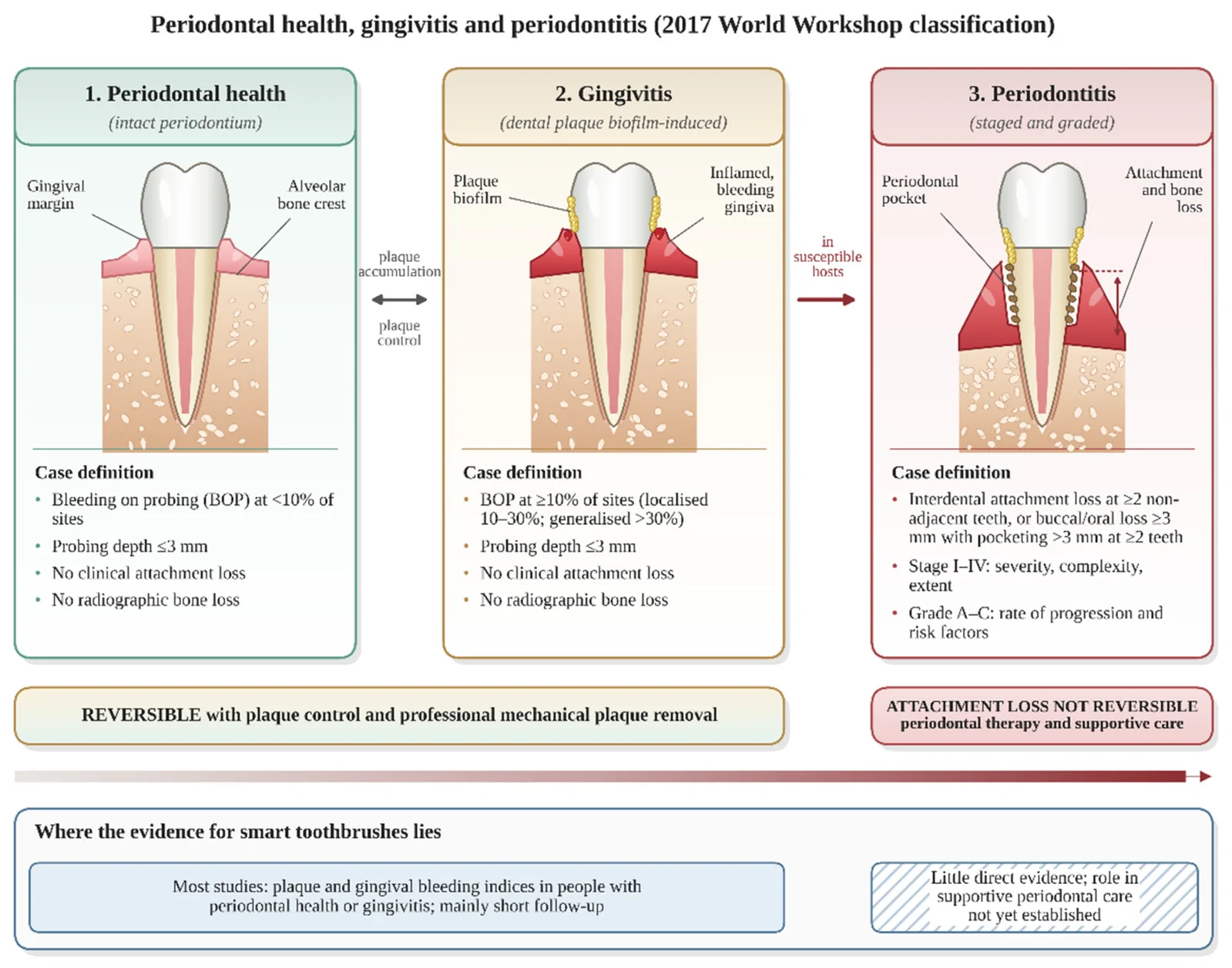
Periodontal health, gingivitis, and periodontitis according to the 2017 World Workshop classification [4,5,6]. Periodontal health on an intact periodontium: bleeding on probing (BOP) at fewer than 10% of sites, probing pocket depth (PPD) ≤ 3 mm, and no attachment or bone loss. Dental plaque biofilm-induced gingivitis: BOP at 10% of sites or more, PPD ≤ 3 mm, and no attachment loss; it is reversible with plaque control. Periodontitis: interdental clinical attachment loss (CAL) at two or more non-adjacent teeth, or buccal or oral CAL ≥ 3 mm with pocketing > 3 mm at two or more teeth; it is staged (I to IV) and graded (A to C), and the attachment and bone loss are irreversible, requiring periodontal therapy and supportive periodontal care (SPC). Schematic drawings show plaque biofilm and bleeding at the gingival margin in gingivitis, and pocket formation with attachment and bone loss in periodontitis. Arrows indicate that gingivitis is reversible with plaque control and may progress to periodontitis in susceptible hosts. The lower panel indicates where the current evidence for smart toothbrush systems lies. Schematic drawn by the author in Python 3.13.16; (Matplotlib 3.11.2).

Toothbrushing provides mechanical disruption of dental plaque and is essential for periodontal health [1]. Regular oral hygiene reduces the risk of dental caries, gingivitis, and periodontitis [2,3]. Incorrect brushing technique, inadequate brushing duration, failure to clean interdental areas, and poor patient compliance reduce the effectiveness of plaque removal. Under the 2017 World Workshop classification, dental plaque biofilm-induced gingivitis is an inflammatory condition of the gingiva without attachment loss that resolves with effective plaque control, whereas periodontitis is defined by interdental clinical attachment loss and is described by stage (severity and complexity, I to IV) and grade (rate of progression, A to C) [4,5,6]. Periodontitis affects approximately 20–50% of the global population [7]. It is characterised by irreversible attachment loss and alveolar bone destruction caused by a dysregulated host inflammatory response to the dental biofilm [8]. Persistent biofilm accumulation disrupts the ecological balance of the oral microbiota. These observations emphasise the role of toothbrushing, interdental cleaning, and regular dental examinations in maintaining periodontal health and limiting disease progression (Figure 1) [9,10].

Digital technology allows toothbrushing behaviour to be recorded objectively. Smart toothbrushes record brushing time, frequency, pressure, and motion [11,12]. Trials of these systems have reported improvements in brushing habits, plaque removal, and gingival health [12,13,14,15], and machine-learning analysis of brushing data has been used to generate individualised recommendations [16,17]. Digital tools now available to dental practitioners include smart toothbrushes, AI-assisted systems, telemonitoring platforms, and gamified oral hygiene applications [16,18]. In this review, a smart toothbrush is a manual or powered toothbrush with integrated sensors (motion, position, or pressure) that sends brushing data to a mobile application. Connected refers only to data transfer, for example by Bluetooth. Rule-based feedback uses fixed, manufacturer-defined thresholds, such as a 2 min timer or a pressure limit. The terms AI-assisted and AI-enabled are used only for systems in which machine-learning models classify brushing patterns or adapt feedback to the individual user as data accumulate [16,18,19]. AI-assisted oral hygiene monitoring can also be based on intraoral photographs that patients take at home and that are analysed by AI software; these systems do not involve a toothbrush and are considered separately. The powered toothbrush provides the mechanical action, whereas the smart components add sensing and feedback [11,19].

These technologies address specific unmet needs. Oral hygiene instruction is usually given at the chairside, and its effect tends to fade between visits. Patients cannot see which surfaces they miss, and clinicians have no objective information on home care between recall appointments. This gap matters most in supportive periodontal care, where plaque control and its reinforcement are part of the recommended long-term management of treated periodontitis [20]. Mobile health (mHealth) interventions delivered by text messages or applications have improved plaque and gingival bleeding compared with conventional instruction [21], and teledentistry has improved oral hygiene in orthodontic patients [22]. Earlier reviews have focused mainly on the technical features of AI-based smart toothbrushes [16] or on AI across oral health care and periodontology [18,19,23]. The present review differs in three ways: it applies a consistent distinction between connected, rule-based, and adaptive AI systems; it separates plaque and gingival outcomes from periodontitis-related outcomes; and it considers the funding of the underlying trials. Its aims were to classify smart and AI-assisted oral hygiene technologies, to summarise the clinical evidence on plaque control, gingival health, oral hygiene behaviour, and periodontitis-related outcomes, and to identify the limitations of this evidence and the barriers to clinical use.

## 2. Review Methodology

This narrative review was prepared with reference to the SANRA (Scale for the Assessment of Narrative Review Articles) checklist [24]. It followed a structured narrative approach, with transparent literature identification, critical appraisal, and conceptual synthesis. A targeted, non-systematic literature search was performed in PubMed/MEDLINE, Scopus, Web of Science, the Cochrane Library, and Google Scholar, and was complemented by manual searches of the reference lists of key publications. The search covered publications from January 2012 to February 2026, when the last search was performed. This period reflects the time in which smartphone health applications and app-connected toothbrushes came into use; earlier evidence on manual and powered toothbrushing was taken mainly from systematic reviews and is cited only as background. Search terms combined oral hygiene and periodontal concepts (“oral hygiene”, “plaque control”, “gingivitis”, “periodontal disease”) with technology-related concepts (“smart toothbrush”, “connected toothbrush”, “AI toothbrush”, “artificial intelligence”, “telemonitoring”, “mHealth dentistry”), using Medical Subject Headings (MeSH) and free-text terms with Boolean operators.

Because the objective of this review was conceptual synthesis and critical interpretation rather than quantitative evidence aggregation, no formal systematic review methodology or meta-analysis was undertaken. Protocol registration, formal risk-of-bias assessment, and a study-flow diagram were therefore not considered appropriate. Publications were selected by the author according to their relevance to the review question, their scientific quality, and their contribution to understanding the clinical role of smart and AI-assisted oral hygiene technologies. Preference was given to randomised controlled trials, other clinical and observational studies, systematic reviews, meta-analyses, and consensus reports addressing plaque control, gingival health, periodontal outcomes, oral hygiene behaviour, telemonitoring, or patient-reported outcomes. Only publications in English were considered, and conference abstracts were not included. Technical studies focusing on algorithm development without clinical outcomes were included only when they helped to explain current capabilities or limitations [25]. The purpose of the review was not to summarise every published device or application, but to interpret the current evidence in a way that separates improvements in oral hygiene from effects on periodontal disease.

Primary studies in humans that evaluated a smart, connected, app-based, or AI-assisted oral hygiene intervention and reported clinical, behavioural, or user-reported outcomes are summarized in this review. Reviews, technical validation studies, and trials of non-connected powered toothbrushes were not tabulated but are discussed where relevant. Table 1 summarises device categories using systematic reviews and representative trials. In total, 58 publications are cited. Design, population, follow-up, intervention and comparator, principal finding, and funding source were also extracted. Funding was classified as industry, non-industry, or not reported. The findings were then grouped by outcome domain: plaque and gingival indices, oral hygiene behaviour and adherence, periodontitis-related clinical outcomes, and user perceptions. Within each domain, the type of technology (connected, rule-based, or adaptive AI) and the comparator were considered, because these determine which part of any effect can be attributed to the smart or AI component.

## 3. Advances in Toothbrushing Technologies

### 3.1. Manual Toothbrushes

Manual toothbrushes are commonly used for plaque control, but effective plaque removal depends on correct brush angulation, controlled pressure, adequate brushing time, and complete tooth-surface cleaning [26,27]. Many individuals do not follow these recommendations during routine toothbrushing, which can lead to plaque accumulation and gingival inflammation. Oral hygiene outcomes also depend on patient motivation, manual dexterity, and oral hygiene knowledge, resulting in variation in brushing effectiveness between individuals.

### 3.2. Powered Toothbrushes

Powered toothbrushes were introduced to reduce the effect of variations in individual brushing technique by using oscillating-rotating or sonic motion. Many devices include timers, pressure sensors, and ergonomic handles to improve brushing performance and reduce excessive brushing force [28]. Randomised trials and systematic reviews have shown better plaque removal and greater improvement in gingival inflammation than with manual toothbrushes, particularly in individuals with inadequate brushing technique [29,30,31]. A network meta-analysis found high certainty for a small benefit of powered over manual toothbrushes for plaque removal, with a very small additional benefit of oscillating-rotating over sonic brushes [32]. Similar findings have been reported in children, adolescents, and older adults [33,34,35]. Powered toothbrushes are also useful for individuals with limited manual dexterity, orthodontic appliances, or difficulty maintaining proper brushing habits [26,36], including people with intellectual disability, for whom one study reported that augmented-reality-guided smart toothbrush training produced greater reductions in debris and calculus indices than conventional visual instruction [37]. The higher cost of powered toothbrushes and their lack of real-time feedback on brushing behaviour have encouraged the development of smart toothbrush systems. Table 1 classifies these digital oral hygiene technologies by mechanism, clinical benefit, and evidence maturity.

**Table 1:** Classification of Digital Oral Hygiene Technologies by Mechanism, Representative Evidence, and Evidence Maturity.

Technology Category	Core Mechanism & Function	Representative Examples/Devices	Primary Clinical Benefit (vs. Predecessor)	Limitations	References
Manual Toothbrush	User-controlled mechanical plaque disruption via bristle friction. Efficacy depends heavily on technique (e.g., Modified Bass).	Standard soft/medium bristle brushes.	Baseline standard for plaque removal when correct technique is applied; low cost and high accessibility.	High dependence on user dexterity, technique, and compliance; inconsistent plaque removal in hard-to-reach areas (e.g., lingual surfaces).	[2,27]
Powered Toothbrush	Automated mechanical action (Oscillating-Rotating [OR] or High-Frequency Sonic [HFS]) to disrupt biofilm. Reduces reliance on user skill.	Non-connected OR (e.g., Braun Oral-B, Procter & Gamble, Cincinnati, OH, USA) or HFS (e.g., Philips Sonicare) devices, (Philips Oral Healthcare, Bothell, WA, USA).	Superior plaque reduction (11% short-term, 21% long-term) and gingivitis reduction (6–11%) compared to manual brushing; particularly effective for OR technology.	Higher cost; does not inherently monitor compliance or provide real-time behavioral feedback; mechanical reliability issues in some models.	[26,30]
Sensor-Based “Smart” Toothbrush	Integrated motion/pressure sensors + Bluetooth connectivity to mobile app. Provides real-time visual feedback on coverage, pressure, and duration.	Connected OR or Sonic systems (e.g., Oral-B iO, Philips Sonicare DiamondClean Smart).	Improves immediate brushing behavior and surface-specific cleaning; real-time feedback enhances user awareness of neglected areas.	Primarily relies on rule-based alerts (timers/pressure); limited adaptive learning; added clinical benefit over standard powered brushes not clearly established.	[38,39]
AI-Driven Monitoring System	Machine-learning algorithms analyze longitudinal brushing patterns to generate personalized, adaptive recommendations. Often includes remote professional monitoring.	Connected toothbrush with proprietary AI position detection (e.g., Oral-B iO9) plus targeted professional messaging; AI analysis of patient-taken intraoral photographs (e.g., DentalMonitoring, (DentalMonitoring SAS, Paris, France).	Identifies recurring neglect patterns; supports individualised coaching beyond fixed rules; improved periodontitis-related outcomes in two short-term trials; photo-based monitoring improved plaque and gingival indices in orthodontic patients.	Preliminary clinical evidence limited to short-term trials; distinction between adaptive AI and fixed-rule algorithms often unclear in commercial marketing.	[13,17,40]
Telemonitoring & App-Integrated	Software-based remote coaching combined with sensor data transmission to dental professionals for personalized feedback.	Interactive telemonitoring toothbrush with professional review; teledentistry platforms.	Enhances adherence through professional accountability; significant reduction in plaque and improvement in hygiene scores compared to manual/standard powered brushes.	Requires infrastructure for professional review; potential privacy concerns; dependent on patient willingness to share data.	[12,38]
App-Only/Software Coaching	Software-based reminders, logging, and educational content without dedicated sensor hardware in the brush. Relies on self-reporting or basic timers.	Standalone oral hygiene coaching apps (e.g., Brush DJ, WhiteTeeth); WhatsApp-based support groups.	Enhances patient engagement and adherence through gamification and reminders; low barrier to entry; improves short-term plaque/gingival indices in orthodontic patients and toothbrushing in young children.	Lacks objective biomechanical data (pressure/coverage); relies on self-reported adherence; effects may diminish over time (novelty effect).	[15,41,42,43]
Gamified Interventions	Reward-based engagement layers (points, badges, music) integrated into apps to motivate behaviour change.	Pediatric-focused app add-ons; Music-based brushing apps (e.g., Brush DJ).	Improves motivation and compliance, particularly in pediatric and adolescent populations; makes oral hygiene routine more enjoyable.	Limited direct evidence for long-term periodontal health outcomes; focus remains on usability/engagement rather than clinical disease modification.	[41,44,45]

### 3.3. Smart Toothbrush Systems

Smart toothbrushes use pressure sensors, motion sensors, tooth-surface mapping, Bluetooth connectivity, and mobile applications to record brushing duration, frequency, pressure, surface coverage, and brush movement [12,16]. These data give objective information on toothbrushing behaviour and reduce dependence on patient recall. Mobile applications provide reminders, visual feedback, progress records, and brushing instructions. In most commercial systems this feedback is rule-based, and only some systems apply machine-learning models to accumulated brushing records to individualise recommendations [16,19]. Smart toothbrush systems have improved plaque removal and gingival health in controlled trials [12,39]. In children, however, a smart toothbrush with an app did not reduce plaque significantly more than electric or manual toothbrushes over 3 months when all groups received oral health education supported by fluorescence plaque imaging [46]. Gingival bleeding recorded through the application has been associated with clinically recorded bleeding on probing, which may support home monitoring and clinical assessment [38]. The characteristics and principal findings of the clinical studies are summarised in Table 2, and their synthesis by outcome domain is presented in Section 7.

## 4. Core Components of Smart Toothbrush Systems

### 4.1. The Four-Component Architecture

**Figure 2:**
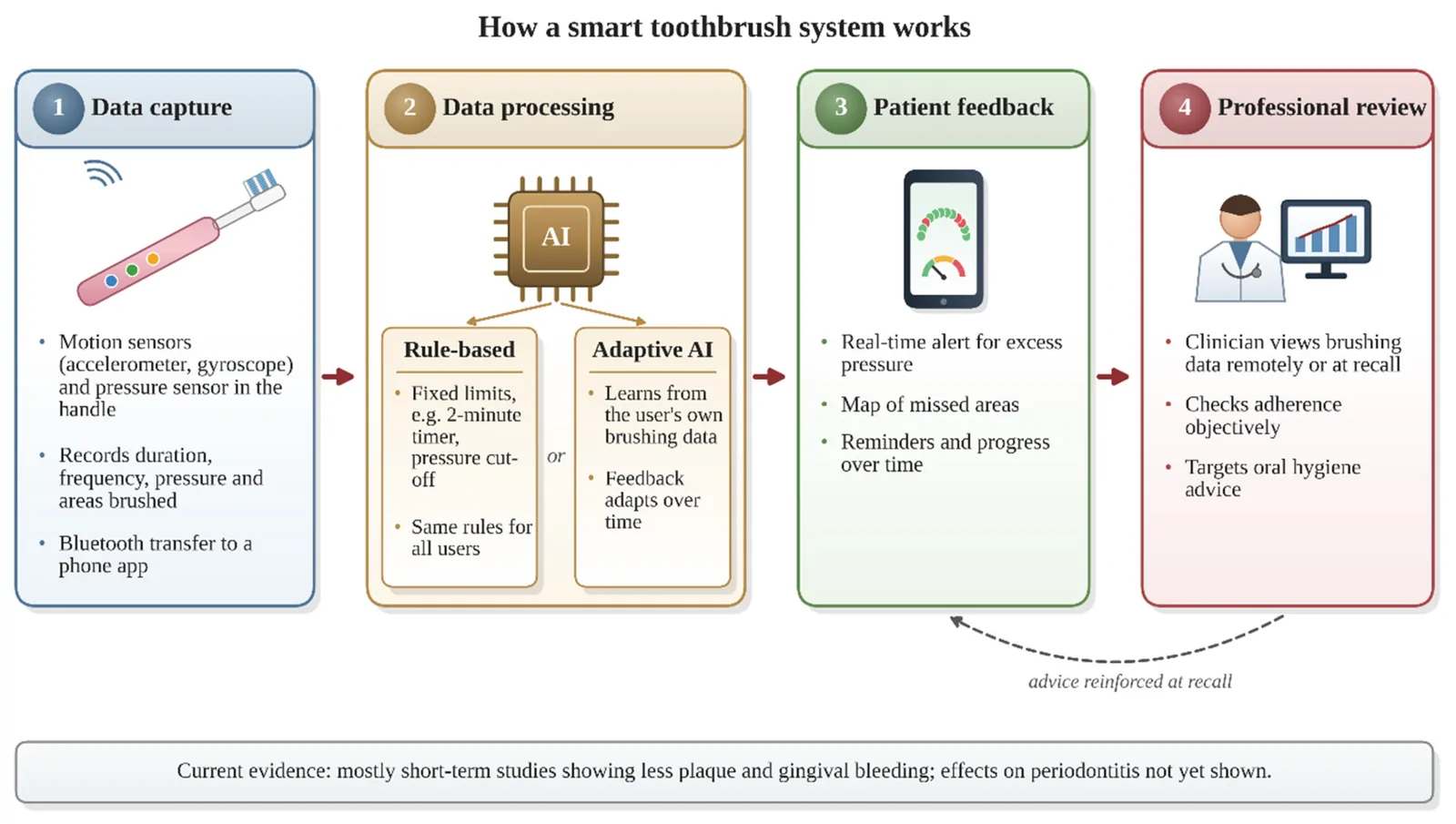
Components of a smart oral hygiene system. Sensor data captured by the toothbrush (1) are processed by fixed rules or, in some systems, by adaptive machine-learning models (2). Feedback is returned to the patient through the application (3), and in telemonitoring models the data are also reviewed by the dental team (4), who reinforce oral hygiene between visits. The dashed arrow indicates that oral hygiene advice is reinforced at recall visits, and the bottom line summarises the current evidence. Drawn by the author in Python (Matplotlib).

A smart oral hygiene system includes four main components: a sensor-equipped toothbrush, a smartphone application, a data analysis module, and a feedback system [17,39,48] (Figure 2). The toothbrush records brushing duration, angulation, pressure, and brush movement in real time [12,38]; in one study, a recurrent probabilistic neural network applied to nine-axis inertial sensor data identified correct Bass-technique brushing positions with over 99% accuracy on smartphone-grade hardware [25]. The smartphone application displays brushing information, identifies missed tooth surfaces, provides reminders, and stores brushing records [39,48]. The data analysis module evaluates repeated brushing records, using either fixed rules or machine-learning models (Section 4.3), and generates recommendations [17]. The feedback system delivers alerts, visual guidance, and coaching messages to improve toothbrushing technique [12,17]. This design provides functions beyond those of a conventional powered toothbrush, which is usually limited to a timer and a pressure alert.

### 4.2. Gamification and Engagement Features

Gamification applies game elements such as points, badges, streaks, music, and animated characters to toothbrushing. It aims to increase motivation, brushing frequency, and, in children, parental participation [14,16,44]. In controlled trials in orthodontic patients, a music-based application and app-based coaching improved plaque or gingival outcomes over 8 to 12 weeks [41,43,50], and in preschool children a smartphone application linked to a sensor-equipped manual toothbrush improved toothbrushing [42]. However, the quality of children’s oral hygiene applications varies considerably [45], and most gamification trials lasted 3 months or less. Interest in rewards may decline once the technology becomes familiar, and none of the studies in Table 2 measured whether gamification maintained brushing quality beyond 6 months. Gamification therefore improves engagement and short-term toothbrushing behaviour, but its effect on periodontal outcomes has not been established.

### 4.3. Rule-Based Analytics Versus Adaptive AI

Rule-based systems compare each brushing session with fixed, manufacturer-defined thresholds, such as a 2 min timer, a pressure limit, or a coverage target for each quadrant. The same rule applies to every user and does not change with use. Adaptive AI systems use machine-learning models trained on sensor data to classify brushing position or technique and to adjust feedback to the individual user as data accumulate [19,25]. An intermediate group uses a machine-learning classifier to detect brush position but then delivers fixed feedback. Many products marketed as AI toothbrushes belong to the rule-based or intermediate groups, and manufacturers rarely disclose how their algorithms work [18,19]. Among the studies in Table 2, a machine-learning component was reported in three: one used a toothbrush with AI position detection [17], and two used AI analysis of intraoral photographs taken by patients at home rather than toothbrush data [13,40]. The others tested connectivity, sensor feedback, or app-based coaching without adaptive learning. Even in the trial by Li et al., the AI component was the proprietary position-detection algorithm of a commercially available connected toothbrush, and the individualised element was targeted messaging from clinicians based on the brushing data [17]. In this review, Bluetooth connectivity, pressure sensing, timers, and predefined alerts are not regarded as AI. This distinction matters clinically, because none of the trials identified compared adaptive AI with well-designed rule-based feedback on the same device.

## 5. Role of AI in Oral Hygiene Monitoring

AI can contribute to oral hygiene monitoring at three levels, and the supporting evidence differs for each. At the level of the single brushing session, machine-learning classifiers applied to inertial sensor data can identify brush position and technique; one development study reported over 99% accuracy in recognising Bass-technique brushing positions [25], and similar approaches underlie the detection of missed surfaces, excessive force, and irregular brushing [18,19]. These are mainly technical validation studies. At the level of the patient over time, AI systems integrate, process, and analyse accumulated data from toothbrush sensors and mobile applications to personalise feedback, prioritise neglected sites, and trigger messages or professional contact [11,19]. Clinical evidence at this level comes from one toothbrush-based trial, in which a commercially available toothbrush with proprietary AI position detection was combined with targeted mHealth messages in patients with periodontitis [17]. A different approach does not use the toothbrush at all: patients take intraoral photographs at home with a smartphone and a scan box, and AI software assesses plaque and gingival status and returns feedback. After non-surgical periodontal therapy, this approach, with or without personalised counselling, improved plaque control and self-care behaviour and was associated with greater reductions in probing pocket depth and clinical attachment level at 3 months [13]. In orthodontic patients, it reduced plaque, gingival inflammation, and white spot lesions compared with standard instruction [40].

The third level, AI in wider periodontal practice, includes automated periodontal charting, radiographic detection of bone loss, and clinical decision support [23,52]. These applications do not depend on toothbrush data and are outside the main scope of this review, although they may eventually be linked with home oral hygiene data (Section 9). At all three levels, AI platforms differ in their underlying technology, and the label AI in a product description does not guarantee adaptive learning (Section 4.3).

## 6. Telemonitoring and Remote Periodontal Prevention

Telemonitoring is the remote transfer of home oral hygiene data to the dental team, who review the data and respond between visits. It differs from app feedback, which is directed only at the patient. In periodontal prevention and supportive periodontal care, telemonitoring can be used to check adherence to recommended brushing frequency and duration, to identify repeated patterns of inadequate cleaning at specific sites, and to trigger timely re-instruction or an earlier recall visit when home care deteriorates or bleeding is reported [12,38]. Because gingival inflammation develops where plaque is left behind, data on site-level coverage are likely to be more useful for telemonitoring than brushing duration or frequency alone. Objective data are also more reliable than patient report: in older adults with mild cognitive impairment, brushing data recorded by the toothbrush correlated poorly with self-reported brushing [49].

The clinical evidence for telemonitoring is still limited. In a 6-month randomised trial, an interactive telemonitoring toothbrush with real-time app feedback and weekly review by a dental professional reduced plaque more than an oscillating-rotating power toothbrush and a manual toothbrush [12]. In a supportive periodontal care population followed for about one year, bleeding on brushing reported through the app in the two weeks before the recall visit, together with the number of residual pockets, predicted bleeding on probing at that visit, which suggests that remote bleeding reports could help identify patients who need earlier review [38]. In patients with stage II or III periodontitis, targeted mHealth micromessages linked to an AI-enabled toothbrush improved control of periodontal inflammation compared with standard care [17]. Beyond toothbrush-based systems, a meta-analysis of 15 randomised trials found that mHealth interventions using text messages or applications improved plaque and gingival bleeding, although heterogeneity was very high [21], and teledentistry education improved plaque and bleeding scores in orthodontic patients [22]. Remote monitoring can also use intraoral photographs taken by patients at home and analysed by AI, which improved oral hygiene outcomes in two trials [13,40]. Remote contact can therefore improve short-term oral hygiene outcomes. No study has yet shown that telemonitoring reduces periodontitis recurrence or tooth loss during long-term supportive periodontal care.

## 7. Clinical Impact on Plaque Control and Periodontal Health

### 7.1. Plaque and Gingivitis Reduction

Greater reductions in plaque and gingivitis have been reported with a new oscillating-rotating toothbrush (Oral-B iO) than with a manual toothbrush over 8 weeks; app feedback was not described in that trial, so the result reflects the powered brush rather than its smart functions [29]. A smart-connected oscillating-rotating toothbrush also reduced gingivitis and plaque more than a smart-connected sonic toothbrush over 6 months [39]. In adolescents, a Bluetooth-enabled interactive toothbrush improved plaque removal and brushing duration, whereas manual toothbrush users showed minimal improvement [47]. A telemonitoring toothbrush with professional review reduced plaque more than power and manual toothbrushes over 6 months [12]. App-based coaching without sensor hardware improved plaque or gingival outcomes in orthodontic patients over 8 to 12 weeks compared with usual care or conventional instruction [41,43,50], and AI analysis of patient-taken intraoral photographs reduced plaque and gingival inflammation in orthodontic patients [40]. Not all comparisons favoured the smart device: in children, a smart toothbrush did not reduce plaque significantly more than electric or manual toothbrushes [46]. Taken together, the controlled trials in Table 2 generally show short-term improvements in plaque and gingival indices. However, in several trials the comparator was a manual toothbrush, so the effect of powered brushing cannot be separated from that of the feedback, and follow-up rarely exceeded 6 months.

### 7.2. Behavioural and Inflammatory Correlates

Smart toothbrush systems may improve oral hygiene behaviour and patients’ perception of their brushing. In a randomised trial comparing a connected with a non-connected electric toothbrush, and in a single-arm study, users of the connected toothbrush reported improvement on several items asking about change in their brushing and oral health, although their perceptions did not correlate with objective brushing or clinical measures [14]. In in-market data from interactive toothbrushes, the proportion of app users reporting gum bleeding fell from 28.8% in the first week to 12.7% in the fifth week, and users brushed longer and with less excessive pressure when using live app feedback [48], and in a supportive periodontal care population self-reported bleeding on brushing was associated with bleeding on probing [38]. These data are useful for monitoring, but they have limitations: in-market data come from self-selected users, bleeding is self-assessed, and associations do not show that the device caused the change. Behavioural and self-reported outcomes should therefore be regarded as supportive rather than confirmatory evidence of clinical benefit.

### 7.3. Periodontitis-Related Clinical Outcomes

Evidence on periodontitis-related outcomes comes from two randomised trials, both in patients with periodontitis receiving treatment. In patients with stage II or III periodontitis, an AI-enabled multimodal-sensing toothbrush combined with targeted mobile health messages, added to guideline-based periodontal therapy, reduced the proportion of inflamed periodontal pockets more than standard care at 6 months (between-group difference 7.9%) [17]. In a three-arm trial, patients who used AI analysis of intraoral photographs taken at home, with or without personalised counselling, after non-surgical periodontal therapy showed better plaque control and self-care behaviour at 6 months and greater reductions in probing pocket depth and clinical attachment level at 3 months than controls; this trial did not use a smart toothbrush [13]. In older adults with mild cognitive impairment, plaque, bleeding on probing, and the proportion of pockets of 4 mm or more improved over 12 months with an app-connected powered toothbrush, even though brushing frequency and duration were below recommendations, but there was no comparator group [49]. The two trials were small (about 100 participants each), lasted 6 months or less, and tested multicomponent interventions, so they do not show which component produced the benefit. In the treated patients, these trials showed short-term improvements in periodontal parameters, but they do not show an effect on the course of periodontitis itself. No study has assessed whether smart toothbrushes prevent the onset of periodontitis, reduce disease recurrence during long-term supportive periodontal care, or reduce tooth loss. At present, these systems are best regarded as adjuncts that may support plaque control during periodontal therapy and maintenance, particularly in patients with recurrent plaque accumulation and gingival inflammation. They do not replace professional periodontal treatment [11,16,17,18].

### 7.4. Industry Sponsorship and Device-Specific Ecosystems

Of the 18 studies in Table 2, six were industry-funded, ten were non-industry funded, and two did not report funding clearly. The industry-funded studies included most trials of branded connected toothbrushes and the large in-market dataset [14,29,38,39,47,48]. Industry funding does not by itself invalidate results, but in these studies the comparator was a manual toothbrush, a competing brand, a non-connected electric toothbrush, or no control group, and controlled follow-up ranged from 2 to 24 weeks. In one of these trials app use was not described, so the smart functions may not have been tested at all [29]. Results obtained within one manufacturer’s device ecosystem may not apply to other products. A further difficulty is that the reported benefits may come from several components acting together: powered brushing itself, sensor-based feedback, mobile application support, behavioural messaging, telemonitoring, and, in a few systems, AI-based personalisation. Powered toothbrushes alone improve plaque removal compared with manual brushing [30,32], so a trial comparing a connected powered toothbrush with a manual toothbrush cannot show the added value of the smart functions.

Only a few trials isolated a single component: connected versus non-connected powered toothbrushes [14], a sensor-equipped toothbrush with versus without an application [42], and AI-assisted monitoring versus standard care, with an additional counselling arm [13]. No trial identified in this review compared adaptive AI with rule-based feedback on the same device. Independent randomised trials with component-controlled or factorial designs, standardised outcome measures, and device-independent comparisons are needed to establish the incremental benefit of each component [29,39,47].

### 7.5. Clinical Significance and Practice Implications

The current evidence supports a limited and specific role for these technologies. Smart toothbrushes may be considered for patients who do not achieve adequate plaque control with standard instruction, patients with limited manual dexterity or intellectual disability, and orthodontic patients, because short-term improvements in plaque and gingival indices or brushing performance have been reported in these groups [37,41,43,50]. In supportive periodontal care, remote brushing and bleeding data may help clinicians identify patients whose home care is declining and adjust re-instruction or recall intervals [12,38]. The size of the benefit should be kept in perspective. Part of the improvement over manual brushing is explained by powered brushing itself [30,32], and evidence for effects on periodontitis-related outcomes is limited to two short trials [13,17]. Device cost, the need for a compatible smartphone, and the time clinicians need to review remote data also influence whether routine use is realistic. These technologies should be used as an adjunct to individual oral hygiene instruction, professional mechanical plaque removal, and periodontal maintenance. They should not be considered a replacement for established periodontal treatment or maintenance protocols.

## 8. Behavioural Change and Patient Compliance

Smart toothbrush systems aim to change behaviour between dental visits. Conventional oral hygiene instruction is given occasionally at the chairside, whereas smart systems give feedback during every brushing session. Several mechanisms may explain their effect: real-time feedback corrects technique as it happens, reminders and streaks prompt regular brushing, progress records support self-monitoring, and awareness that data may be reviewed by a clinician can increase accountability. Theory-based coaching applications that use these mechanisms improved oral hygiene in adolescents [43], and mobile health applications and digital notifications have supported oral hygiene behaviour in orthodontic patients [15,41,50].

Whether these effects last is less certain. Most trials followed participants for 12 weeks or less, and novelty effects are possible, because interest in feedback and rewards may fall once the technology becomes familiar. In-market data come from self-selected users who are probably more motivated than typical patients [48]. Some AI-based systems add real-time plaque detection, but because sensors, applications, messaging, and AI are delivered together, the specific role of adaptive AI in sustaining behaviour cannot be separated from that of the other components [16]. Future studies should report adherence and brushing quality beyond 6 to 12 months and after the active intervention ends.

## 9. Personalized Preventive Periodontal Care

Precision periodontology aims to tailor prevention and treatment to the individual by combining clinical, biological, and behavioural information [53]. Smart toothbrush data are behavioural: they describe how often, how long, and how completely a patient brushes, and some systems add self-reported bleeding. On their own, these data cannot show whether the periodontal tissues are stable. Their value would increase if they were combined with biological information. Inflammatory markers in gingival crevicular fluid, such as matrix metalloproteinase-8 (MMP-8), are associated with pocket depth and periodontitis grade [54], and active MMP-8 can be measured with point-of-care tests [55]. Microbiological and other host-derived data have also been proposed as inputs for data-driven periodontal diagnosis [53]. Mobile health tools used between visits already improve plaque and bleeding scores [21,22], and powered toothbrushes provide an effective basis for home plaque control [32].

A realistic integration model would combine three data streams. The toothbrush and application would provide continuous behavioural data and self-reported bleeding; periodic biomarker or point-of-care tests at recall visits would provide information on inflammatory activity; and clinical examination would provide BOP, PPD, and CAL. An analytical model could then identify patients whose risk of recurrence is rising, for example a patient with declining brushing coverage and rising MMP-8 levels, and prompt earlier professional contact. At present this model is conceptual. No study has combined toothbrush-derived data with biomarker data, the predictive value of such combinations is unknown, and data from different devices and tests are not yet interoperable. Prospective studies should test whether combined behavioural and biological monitoring improves the timing of interventions and long-term periodontal stability.

## 10. Barriers to Implementation

Several barriers currently limit routine clinical use. Device cost: connected toothbrushes and their replacement heads cost more than manual or basic powered toothbrushes, which may widen inequalities in access [16,51]. Digital literacy: older adults and people with cognitive or physical limitations may find applications difficult to use, although they may benefit most [49,56]. Data privacy and security: brushing data, bleeding reports, and linked health information are personal health data, and the terms under which manufacturers store, share, or reuse them are often unclear to patients and clinicians. Interoperability: data are usually held within one manufacturer’s application and cannot easily be transferred to electronic dental records or combined with data from other devices [57]. Algorithm transparency: manufacturers seldom disclose whether feedback is rule-based or AI-driven, how algorithms were trained and validated, or how they perform across age groups and dentitions [18,19]. Industry bias and limited independent evidence: most trials are short, many are industry-funded, and independent long-term evidence is scarce (Section 7.4). Finally, power-brushing, sensors, AI features, and smartphone applications are usually delivered as one product, which makes it hard to quantify the contribution of each component [11,16,17,18].

## 11. Research Agenda and Clinical Translation: Prioritized Steps and Actionable Recommendations

The gaps identified in this review can be ranked by how directly they limit clinical use. The research priorities below are listed in that order, followed by recommendations that clinicians can apply now and steps for manufacturers and developers.

Priority 1 is long-term trials with periodontitis-related outcomes. Independent randomised trials of at least 12 months are needed in patients receiving supportive periodontal care, with outcomes such as the number of residual pockets with bleeding on probing, disease recurrence, need for retreatment, and tooth loss, rather than plaque and gingival indices alone [13,17]. Priority 2 is component-controlled designs. Trials should compare the same powered toothbrush with and without app feedback, with and without professional telemonitoring, and with rule-based versus adaptive AI feedback, so that the incremental benefit of each component can be estimated [14,42]. Priority 3 is independence and transparency. Trials should be investigator-led where possible and prospectively registered, and they should report funding, device settings, and whether feedback is rule-based or based on machine learning.

Priority 4 is sustained adherence. Studies should measure brushing quality and engagement beyond 6 to 12 months and after the active intervention ends, and should report how many participants stop using the device. Priority 5 is equity and cost-effectiveness. Studies should include older adults, people with disabilities, and lower-income groups, and should compare costs and outcomes with those of basic powered toothbrushes and standard oral hygiene instruction [49,56]. Priority 6 is integration with biological monitoring. Prospective studies should test whether combining toothbrush data with biomarkers such as MMP-8 improves the timing of interventions in supportive periodontal care [54,55]. Machine learning, biosensors, teledentistry, and electronic dental records may eventually be combined for periodontal care, but this remains to be tested [52,53,57,58].

Until such evidence is available, the following steps are reasonable in clinical practice. First, smart toothbrushes should be used selectively, as an adjunct, for patients who do not achieve adequate plaque control with standard instruction, patients with limited dexterity or intellectual disability, and orthodontic patients [37,41,43,50]. Second, patients should be told that the expected benefit is better plaque control and less gingival bleeding, not prevention of periodontitis. Third, where patients share brushing or bleeding data, clinicians should review the data at recall and use them to target re-instruction to neglected sites rather than to brushing time alone [12,38]. Fourth, interdental cleaning, professional mechanical plaque removal, and risk-based recall intervals remain the basis of periodontal prevention and supportive care, and smart devices should not displace them [20]. Fifth, before recommending a device, clinicians should check how the manufacturer stores and shares patient data and should obtain the patient’s consent before viewing it.

For manufacturers and developers, the priorities are to state clearly whether feedback is rule-based or AI-driven, to publish validation data for their algorithms, to adopt open data formats that allow export to electronic dental records, and to support independent evaluation of their devices [18,19,57].

## 12. Conclusions

Current evidence suggests that smart toothbrushes and digitally assisted oral hygiene systems may improve plaque control, brushing behaviour, adherence, and short-term gingival outcomes. Two small randomised trials suggest that AI-assisted monitoring may add short-term benefits to periodontal therapy in patients with periodontitis. However, the incremental benefit specifically attributable to AI-based functions and their impact on long-term periodontal outcomes remain to be established. In practice, these systems are best used as adjuncts to oral hygiene instruction and supportive periodontal care, particularly for patients with persistent plaque and gingival inflammation. Independent long-term trials with component-controlled designs are needed before they can be recommended for the prevention of periodontitis.

## Figures and Tables

**Table 2:** Characteristics and principal findings of studies evaluating smart toothbrushes and digitally assisted or AI-assisted oral hygiene systems.

Author/Year	Study Design	Population/Sample	Follow-Up	Intervention/Comparator	Principal Finding	Funding
Grender et al., 2020 [29]	Randomized examiner-blind parallel trial	110 adults with gingivitis	8 weeks	Oral-B iO vs. manual toothbrush	Greater reductions in plaque, bleeding sites, and MGI than manual toothbrush; app use not described	Industry
Goyal et al., 2021 [39]	Randomized open-label parallel trial	110 adults with gingivitis	24 weeks	Oral-B iO vs. Philips Sonicare DiamondClean	Greater reductions in MGI, GBI, bleeding sites, and plaque than connected sonic toothbrush	Industry
Erbe et al., 2018 [47]	Randomized single-blind trial	60 adolescents (13–17 years)	2 weeks	Bluetooth interactive powered toothbrush vs. manual toothbrush	Whole-mouth plaque reduced by 34% vs. 1.7% with manual toothbrush; brushing time increased by 34 s	Industry
Gomez et al., 2025 [14]	Randomized trial and single-arm study	RCT: 80 adults; Longitudinal: 159 adults	6 weeks (RCT); 4–5 weeks (single-arm)	Connected powered toothbrush vs. non-connected powered toothbrush	Better self-rated change in brushing and oral health on some items; perceptions not correlated with clinical measures	Industry
Tonetti et al., 2020 [38]	Clinical audit	100 supportive periodontal therapy patients	Mean 362 days	Connected powered toothbrush; no comparator	App-reported bleeding on brushing and residual pockets predicted BOP at SPC recall; 75% retained	Industry
Thurnay et al., 2022 [48]	Observational app-based study	439,481 users; 16.7 million brushing sessions	2–5 weeks	Interactive powered toothbrushes with app feedback	Self-reported gum bleeding fell from 28.8% (week 1) to 12.7% (week 5); longer brushing with live feedback; no comparator	Industry
Li Y. et al., 2024 [17]	Randomized superiority trial	100 patients with Stage II/III periodontitis	6 months	AI-enabled toothbrush + app + SMS guidance vs. standard care †	Greater reduction in proportion of inflamed pockets than standard care (difference 7.9%)	Non-industry
Hu et al., 2025 [13]	Single-blind randomized trial	98 patients with periodontitis	6 months	AI analysis of patient-taken intraoral photographs (DentalMonitoring), with or without counselling, vs. standard care †	Better plaque control and self-care at 6 months; greater PPD and CAL reduction at 3 months vs. control	Non-industry
Kim et al., 2025 [12]	Randomized controlled trial	150 adults	6 months	Telemonitoring toothbrush; powered toothbrush; manual toothbrush	Greater plaque reduction than OR power and manual toothbrushes	Non-industry
Flyborg et al., 2023 [49]	Observational sub-study	170 older adults with mild cognitive impairment	12 months	App-connected powered toothbrush; no comparator	Plaque, BOP, and pockets of 4 mm or more improved over 12 months; objective data correlated poorly with self-report; no comparator	Non-industry
Sharma et al., 2025 [40]	Prospective clinical study	40 orthodontic patients	3 months	AI analysis of patient-taken intraoral photographs (DentalMonitoring) vs. standard oral hygiene instruction †	Greater reduction in plaque, gingival inflammation, and white spot lesions than control	Non-industry
Alfiya et al., 2025 [41]	Randomized controlled trial	36 orthodontic patients	8 weeks	Brush DJ application vs. usual care	Lower plaque index at 4 and 8 weeks; gingival index lower at 4 weeks only	Non-industry
Sağoğlu et al., 2025 [50]	Randomized clinical trial	60 orthodontic patients	3 months	Dentabuddy application vs. conventional oral hygiene training	Greater gingival index improvement in fair/poor hygiene subgroups; better brushing technique	Non-industry
Alkilzy et al., 2019 [42]	Prospective, controlled, single-blinded RCT	49 preschool children (mean age 5.1 ± 0.6 years)	12 weeks	Manual toothbrush with gravitation sensor + smartphone app vs. manual toothbrush with sensor only (no app)	Better toothbrushing with app than with sensor alone	Not reported
Scheerman et al., 2020 [43]	Parallel-group, single-blinded RCT	132 adolescents (12–16 years) with fixed orthodontic appliances	12 weeks	“WhiteTeeth” mobile app (theory-based coaching) + usual care vs. usual care only	Less gingival bleeding at 6 weeks and less plaque at 12 weeks than usual care	Non-industry
Lee et al., 2023 [46]	Randomized clinical trial	68 children (6–12 years)	3 months	Smart toothbrush+ app vs. electric toothbrush vs. manual toothbrush (plaque assessed by QLF imaging)	Plaque decreased with smart toothbrush, but no significant difference between the three groups	Non-industry
Jeon et al., 2021 [37]	Non-equivalent control group pre-test–post-test design	30 adults with intellectual disability in residential care	12 weeks	AR-based smart toothbrush training (“Brush Monster”) vs. visual material training	Better brushing performance and oral hygiene (debris and calculus indices) than visual training	Non-industry
Selvakumar et al., 2025 [51]	Cross-sectional survey	101 dental students and professionals	Single assessment	Questionnaire on awareness and attitudes	Survey of awareness and attitudes; no clinical outcomes	Not reported

Footnote: Funding categories: Industry, direct manufacturer funding or author affiliation with a manufacturer; Non-industry, academic or governmental funding, investigator-initiated study, or no external funding declared (some of these studies used commercial devices, but the authors declared that manufacturers had no role in study design, analysis, or reporting); Not reported, funding source not stated or unclear. † A machine-learning (AI) component was reported. BOP, bleeding on probing; CAL, clinical attachment level; GBI, gingival bleeding index; MGI, modified gingival index; OR, oscillating-rotating; PPD, probing pocket depth; QLF, quantitative light-induced fluorescence; SPC, supportive periodontal care.

## Data Availability

No new data were created or analyzed in this study. Data sharing is not applicable to this article.

## Abbreviations

The following abbreviations are used in this manuscript:

Abbreviation	Full Form
AI	Artificial Intelligence
AR	Augmented Reality
BOP	Bleeding on Probing
CAL	Clinical Attachment Level
HFS	High-Frequency Sonic
MeSH	Medical Subject Headings
mHealth	Mobile Health
MMP-8	Matrix Metalloproteinase-8
OR	Oscillating-Rotating
PPD	Probing Pocket Depth
QLF	Quantitative Light-Induced Fluorescence
RCT	Randomized Controlled Trial
SANRA	Scale for the Assessment of Narrative Review Articles
SMS	Short Message Service
SPC	Supportive Periodontal Care
